# Writing about health inequality: recommendations for accurate and impactful presentation of evidence

**DOI:** 10.1186/s12939-025-02548-6

**Published:** 2025-09-02

**Authors:** Nicole Bergen, Katherine Kirkby, Aluisio J D Barros, Paula Braveman, Peter Goldblatt, Theadora Swift Koller, Oscar J Mujica, Devaki Nambiar, Owen O’Donnell, Anne Schlotheuber, Vivian Welch, Ahmad Reza Hosseinpoor

**Affiliations:** 1 Department of Data, Digital Health, Analytics and AI, World Health Organization, 20 Avenue Appia, Geneva 27, CH-1211 Switzerland; 2 International Center for Equity in Health, Universidade Federal de Pelotas, Pelotas, Brazil; 3 Center for Health Equity, School of Medicine, University of California, San Francisco, United States of America; 4 Institute of Health Equity, University College London, London, UK; 5 Department of Gender, Rights, Equity & Sexual Misconduct Prevention, World Health Organization, 20 Avenue Appia, Geneva 27, CH-1211 Switzerland; 6 Social Epidemiology, Department of Evidence and Intelligence for Action in Health, Pan American Health Organization, Washington, DC United States of America; 7 Erasmus School of Economics, Erasmus School of Health Policy and Management, Erasmus University Rotterdam, Rotterdam, the Netherlands; 8 Bruyère Health Research Institute and School of Epidemiology and Public Health, University of Ottawa, Ottawa, Canada

**Keywords:** Health inequality, Health equity, Analysis, Reporting, Writing, Best practices, Terminology, Language use, Affirming language, Statistics

## Abstract

Health and development agendas and programmes often prioritize the reduction of unfair and remediable health inequalities. There is a growing amount of data pertaining to health inequalities. Written outputs, including academic research papers, are key tools for describing health inequalities. Epidemiologists, data analysts, policy advisors and health equity scholars can have greater impact through accurate, concise and compelling presentation of this evidence and so assist those advocating for action to close health gaps. We make recommendations to improve the accuracy and impact of written evidence on health inequality. Focusing on the micro, macro and meta aspects of developing written reports, we drew from our varied experiences promoting health inequality monitoring to identify key strategies specific to this field, which were further expanded and explored through literature searches and consultation with experts. We recommend four general strategies: (i) using terminology deliberately and consistently; (ii) presenting statistical content accurately and with sufficient detail; (iii) adhering to guidelines and best practices for reporting; and (iv) respecting and upholding the interests of affected communities. Specifically, we address the use of terminology related to health inequality and health inequity, dimensions of inequality and determinants of health, economic inequality and economic-related inequality, sex and gender, and race and ethnicity. We present common pitfalls related to reporting statistical content, underscoring the importance of clarity when reporting association and causation. We advocate for engaged and inclusive writing processes that use affirming language and adopt strength-based messaging. This guidance is intended to increase the impact of written evidence on efforts to tackle avoidable health inequalities.

## Introduction

Since the 1960s, the number of scientific publications on health inequality has grown exponentially, outpacing growth in scientific publications in general [1]. There is also greater availability and accessibility of data about health inequalities. This increased interest in characterizing and addressing health inequalities is also evident in major health and development initiatives, which increasingly prioritize tackling health inequality and embedding this in their monitoring approaches [2]. Monitoring and speeding progress toward the goal of reducing avoidable health inequalities requires accurate and impactful reporting of the evidence [3].

Analyses of health inequality use disaggregated data to describe differences in health between population subgroups defined by demographic, geographic or socioeconomic characteristics that are historically associated with social advantage or disadvantage [4]. Health inequality measurements serve as metrics through which to assess health *inequity* – differences in health between groups of people that are unfair, avoidable or remediable [4, 5]. The effective dissemination of evidence on health inequalities can contribute to broader global and local efforts to improve health status, with the aim of improving the lives of populations experiencing disadvantage and, thus, reducing health inequities. When communicated appropriately to the relevant audience, at the opportune time and in the right format, high-quality evidence on health inequality can guide health and other relevant policies, programming and practices designed to address health inequity [4].

Compelling and accurate written reports on health inequality analyses provide insight into the nature of health inequities and can inform actions to ameliorate them. However, common pitfalls of reporting lessen accuracy and weaken impact. For example, ambiguous terminology and too little (or too much) statistical detail obscure interpretation of evidence. While guidance for analyzing and interpreting health inequalities [4, 6, 7], reporting on health data [8] and writing academic articles [9, 10] are readily available, there is little guidance specific to the reporting of health inequality analyses.

This paper draws on varied experiences in health equity research to recommend strategies to help scholars and advocates write up the results of health inequality analyses with greater accuracy, consistency and impact, while also fostering more inclusive and respectful writing practices. We aim to encourage more robust and effective reporting of evidence on health inequalities that makes their unfairness more evident and actionable.

## Approach

Our recommendations were informed by practical experiences with health inequality analyses. Some of the coauthors are part of the World Health Organization (WHO) Health Inequality Monitoring team. Others bring experience of presenting policy-directed evidence on health inequality.

Drawing on our perspectives and experiences, as well as academic and grey literature identified through focused searches on PubMed and Google Scholar, we identified key strategies for reporting evidence on health inequality. These strategies encompass the “micro” (relating to language choice, terminology and technical detail), the “macro” (relating to structure, format and flow) and the “meta” (relating to the processes of planning and carrying out writing projects). We focused on writing about health inequality analyses. Many of the ideas, however, are applicable to other formats for reporting evidence on health inequality. The paper does not aim to be comprehensive. It complements general guidance on scientific writing.

## Cross-cutting strategies

We identified four overarching strategies to enhance the accuracy and impact of written reports on health inequality analyses. Two reflect micro aspects of writing: (i) deliberate and consistent use of terminology; and (ii) presentation of statistical content accurately and with sufficient detail. One reflects macro aspects of writing: (iii) alignment of written outputs with other relevant reporting standards and guidelines. And one reflects the meta process of writing: (iv) adoption of inclusive and respectful writing practices. We applied each strategy to the reporting of health inequalities.

### Micro strategies

Terminology and statistical analyses are the building blocks for the written presentation and interpretation of evidence on health inequalities.

#### Strategy 1: Use terminology deliberately and consistently

We highlight common sources of confusion arising from the use of terminology in health inequality analyses, and we recommend practices to avoid these pitfalls and promote conceptual clarity. While there are particular considerations relevant to the measurement and categorization of any inequality dimension [4], special consideration is given to sex and gender (subsection D) and race and ethnicity (subsection E).

##### A. Health inequality and health inequity

Differences in health between groups of individuals categorized by some dimension may be referred to with numerous labels: health inequality, health disparity, health gradient, health gap, social inequality in health, and health inequity [11, 12]. Each term may carry a distinct and specific meaning to some readers, and different meaning for others [13–16]. An important distinction is between health inequality and health inequity [13]. The WHO defines the first as a measured difference in health between population subgroups. Health inequalities can be measured and monitored over time. For the past three decades, the term has been used globally to refer to health differences associated with social advantage and disadvantage [4]. The WHO definition of health inequity refers to unfair, avoidable or remediable differences in health between groups of people. In some cases, the absence of a difference between groups (i.e. a situation of equality) might be considered inequitable [4, 17]. Panel 1 demonstrates how the two terms may be used effectively as part of reporting.

**Table:** 

**Panel 1 Health inequality and health inequity**	
When presenting measurements of health inequality and discussing their implications for health equity, an effective approach is to explicitly distinguish between the two concepts. This may entail presenting the results of inequality analysis (e.g. as part of the results section of a manuscript) and then discussing the supporting evidence and assessments needed for inequalities to be considered inequitable (e.g. as part of the discussion section of a manuscript). Discussion of the concept of health inequity requires contextualization to clarify how evaluations regarding fairness and justice are made, acknowledging that different value judgements may lead to different conclusions regarding equity. The following sentences demonstrate less effective and more effective approaches to writing about health inequality and health inequity as part of a hypothetical analysis:	
Less effective: *The analysis shows a large inequality in health service coverage – a 20% point difference between the richest and poorest groups – that indicates inequity favouring the richest due to greater access to resources.* More effective: *The analysis measured inequality in health service coverage by economic status and revealed higher coverage among richer subgroups. The level of coverage was 20% points higher in the richest group than in the poorest group. Supporting evidence showed the inequality was due to richer groups having greater access to healthcare resources that higher income made more affordable. Those considering such inequality in access to be unfair will view the inequality in health service coverage as inequitable.*	

Inconsistencies in the meanings attached to labels for health differences contribute to fragmentation of this field of research [18, 19]. It is outside the scope of this paper to resolve these debates or offer definitive guidance on the use of terminology. Rather, in the absence of a clear consensus, we recommend the use of language that best reflects the customary usage and preferences of the target audiences, and that terminology always be clearly defined and used consistently.

##### B. Dimensions of inequality and determinants of health

The terms “dimensions of inequality” and “determinants of health” are prone to confusion or conflation. While the two terms can describe common or similar factors associated with health, their respective meanings and connotations differ.

Dimensions of inequality (sometimes termed equity stratifiers) refer to the criteria upon which population subgroups are defined when measuring health inequalities, such as demographic, socioeconomic and geographical characteristics [4]. In the interest of using health inequality monitoring to advance health equity, dimensions of inequality encompass characteristics that are reasonably likely to reflect unfair differences between population subgroups. Frameworks such as EQUALSS GUIDE Multiple [20] and PROGRESS-Plus [21] provide structured lists of dimensions of inequality that are commonly applied (Table 1). Because people’s experiences of health are shaped by multiple, different characteristics, studying and reporting inequality requires the consideration of multiple dimensions of inequality [20].

**Table 1:** Frameworks specifying common dimensions of inequality

Framework	Dimensions of inequality
EQUALSS GUIDE Multiple framework [20]	ethnicity and race; qualifications and education; underserved areas; age; language and religion; sex; sexual orientation; gender identification; underrepresented groups (inclusion groups); income and wealth; disability (physical, mental and learning); employment and occupation; multiple disadvantage
PROGRESS-Plus framework [21]	place of residence; race/ethnicity/culture/language; occupation; gender/sex; religion; education; socioeconomic status; social capital; additional context-specific dimensions (“Plus”) such as age, disability, immigration/citizenship status, insurance status and sexual orientation

WHO describes determinants of health as factors that affect the health of individuals and communities. They can include factors related to the social and economic environment and the physical environment, and they may reflect social context and position that is based on an individual’s characteristics and behaviours [4, 22]. Evidence about determinants of health is often provided as part of the background of health inequality reports, as well as when discussing the potential drivers of inequalities and perpetuating factors. Moreover, at times it is important to monitor inequalities not only in health but in the factors that are the most important influences on health. Therefore, determinants of health may be a focal topic for health inequality monitoring, such that data pertaining to the determinants of health can be disaggregated according to relevant dimensions of inequality to quantify and track the extent of inequality over time [4].

Panel 2 presents examples of dimensions of inequality and determinants of health indicators that are featured in the WHO Health Inequality Data Repository, noting where certain factors overlap.

**Table:** 

**Panel 2 Dimensions of inequality and determinants of health featured in the WHO Health Inequality Data Repository**	
The WHO Health Inequality Data Repository is the largest repository for datasets of disaggregated data, covering a diversity of health and health-related topics, dimensions of inequality and populations [23, 24]. As of 2025, the Repository featured 24 dimensions of inequality, such as age, economic status, education level, ethnicity, place of residence, poverty status, sex and subnational region.	
The Repository contains numerous datasets with information on health determinants indicators, including indicators related to education, communication, employment, the environment, income and poverty, household characteristics and material deprivation.	
Certain characteristics are included as both dimensions of inequality and determinants of health. For example, education is used as a dimension of inequality in immunization, HIV/AIDS, and reproductive, maternal and child health indicators, among others. Health indicators in those topics are disaggregated by education, which is usually measured according to the level of schooling completed, and categorized into primary, secondary and tertiary (noting that in some cases, the categories are divided more or less finely). Education is also featured as determinant of health indicators. For example, the indicators related to education include “percentage of people with secondary or higher education” and “net attendance rate of primary school.” Health determinant indicators related to education are disaggregated by dimensions of inequality such as marital status, place of residence and subnational region.	

##### C. Economic inequality and economic-related inequality in health

There is an important distinction between economic inequality and economic-related (or economic-based) inequality in health^1^. Economic inequality pertains to the distribution of economic resources, e.g., wealth or income, in a population [25]. Economic inequality can be measured using a variety of metrics. For instance, the Gini coefficient can be used to measure the extent to which the distribution of income in a population deviates from an entirely equal distribution in which everyone has the same income.

Economic-related inequality in health refers to variation in health between subgroups (or individuals) of different economic status. An exploration of economic-related inequality in healthcare coverage might compare coverage of the rich versus coverage of the poor. If the richer groups have better coverage than the poorer ones, then there is a pro-rich inequality, or a pro-rich gradient or pattern in coverage [6]. If poorer groups were to report higher coverage than richer groups, then there would be pro-poor inequality in coverage (in that case, since poorer groups may also have greater need for healthcare, additional context may be required when interpreting and presenting findings).

Although measures of economic inequality are distinct from measures of economic-related inequality in health, the former may be relevant as part of exploring the state of inequality in a health topic. Economic inequality measures can provide important background information and context about the population. Further, the associations between economic inequality and health indicators may serve as a supplementary analysis.

##### D. Sex and gender

Sex and gender are common dimensions of inequality explored through health inequality analyses. They are distinct concepts, although they are often conflated or used in a manner that does not reflect how the variable was measured in the underlying data. Sex generally refers to “different biological and physiological characteristics^2^ of females, males and intersex persons, such as chromosomes, hormones and reproductive organs” [26]. Sex is a classification assigned at birth which, except in specific circumstances, usually corresponds with the sex or gender identity recorded on official documents (e.g. identity cards, passports and birth certificates) [27]. Increasingly, however, a distinction is drawn between sex assigned at birth and gender identity, referring to a deeply felt, internal and individual sense of one’s own identity with respect to gender [4].

Gender is a social construction that reflects the norms, stereotypes, and roles expected of people according to their assigned sex (i.e. boys, girls, men, and women) and which often underpin inequalities in access to opportunities, capacities and resources. Gender identities of individuals do not always correspond to these norms and may change over time and in response to contexts. Considerations should be given to what aspects of gender are relevant to a given research question when collecting, categorizing and analyzing information about gender as a dimension of inequality [28, 29].

When writing about health inequalities, accurate descriptions of sex, gender and gender identity variables are needed to ensure transparency surrounding the aspect of health inequality being reported [30]. Ideally, health inequality reports should include information about how sex and gender data were collected to permit accurate interpretation of results [31]. In some cases, the corresponding variable may be described as “sex/gender.”^3^ While routine collection of data on gender identity is a growing, it is still far from routine and “gender” is sometimes assumed to be a shorthand for gender identity. Care should be taken with any approach that may lead to conflation of the distinct concepts. If the distinction is not evident or if further clarification is not possible, this should be acknowledged.

##### E. Race and ethnicity

Both race and ethnicity are social constructs that refer to social groups, “often sharing cultural heritage and ancestry, that are forged by oppressive systems of race relation” [12]. Race is a concept that groups people based on observable physical features, such as skin colour or hair texture, noting that it is a social construct that does not correspond to any permanent or discrete biological subspecies or categories^4^ [34]. Ethnicity is commonly understood to be based on perceived common ancestry, history and cultural practices [34]. Race, ethnicity or race/ethnicity constitute important dimensions of inequality, especially for national and subnational analyses, as they reflect forms of discrimination that, in most places, coincide with aspects of health and health determinants. These dimensions of inequality have more limited applications for global comparisons, as the categorization of these factors are difficult to standardize and compare across countries.

The connotations and terminologies assigned to “race”, “ethnicity” or “race/ethnicity” are specific to the context in which they are used [35]. In many contexts, race has been used as “a symbolic category [actively created and recreated, rather than pre-given], based on phenotype or ancestry and constructed to specific racial and historical contexts, that is misrecognized as a natural category” [36, 37]. The practice of using race as a biological construct can result in harms for historically disadvantaged groups and exacerbate inequalities^5^ [36]. It is recognized that the concept of race has social meaning “both as self-expressed group identity (for example, as part of the Black Lives Matter movement that has resonated across the globe) and in the ways that discrimination and racism have violated human rights and denied people equal opportunities and outcomes” [34].

Panel 3 provides examples of how race and ethnicity have been understood and presented in the contexts of the United States of America and Brazil, both countries with histories of racialized colonialism and changing ethnic diversity.

**Table:** 

**Panel 3 Concepts of race and ethnicity in the U nited States of America and Brazil**	
Federal agencies in the United States of America use standards that identify five categories for data on race (American Indian or Alaska Native, Asian, Black or African American, Native Hawaiian or Other Pacific Islander, and White) and two categories for data on ethnicity (“Hispanic or Latino,” and “Not Hispanic or Latino”), noting that categories are “socio-political constructs and should not be interpreted as being scientific or anthropological in nature” [38]. A 2020 policy by the American Medical Association explicitly called for “ending the practice of using race as a proxy for biology in medical education, research and clinical practice” [36].	
In Brazil, there have been evolving approaches to categorizing ethnicity and race, or color/race as used in the Brazilian Demographic Census. The current terms used to describe color/race are *Brancos* (White people), *Pardos* (people of mixed race), *Pretos* (Black people), *Amarelos* (East Asian people) and Indigenous Peoples [39]. Pretos and Pardos are often merged in a category of Negros (which does not have the same negative connotation as elsewhere) for purposes such as quotas for entry at public universities, a policy established in 2012 by a federal law. The National Policy for Comprehensive Health of the Black Population (*Política Nacional de Saúde Integral da População Negra*), established by the Brazilian Ministry of Health, is an example of a policy intervention that addresses the legacy of racialized colonialism [40]. It lays out affirmative actions and strategies to promote racial equality, guided by the use of racial classifications for priority-setting and decision-making.	

#### Strategy 2. Present statistical content accurately and with sufficient detail

As in any writing about statistical analysis, statistical content should be presented accurately and with sufficient detail, appropriately highlighting limitations. Reporting the methods and results of health inequality analyses often entails explanation and interpretation of measures of health differences between two or more subgroups, which may be expressed as a single number in a summary measure [7].

##### A. Units of measurement

Reporting and comparing results of inequality analyses requires attention to the units of measurement. This is important because the unit of a summary measure of health inequality – and the interpretation of findings – may not be intuitive for all audiences. Measures of absolute inequality, which show the magnitude of difference in health between subgroups, retain the same unit of measurement as the health indicator. On the other hand, measures of relative inequality, which show difference in shares of total health of subgroups or ratios of health of the subgroups, are unitless. The most common summary measures of health inequality have been summarized elsewhere, including their units of measure and interpretation [7].

A common point of confusion in some health inequality reports surrounds the use of the unit *percentage* and *percentage point.* The issue may arise when reporting includes measures of absolute inequality (such as difference) for a health indicator expressed as a percentage. While percentage expresses a proportion out of 100, percentage point is the appropriate unit when making an absolute comparison between two percentages. It is correct, for example, to state that an increase in health service coverage from 50 to 55% represents an increase of 10% or 5 percentage points.

The units of summary measures of inequality should be defined and interpreted, including the value corresponding to no inequality, correspondence between the sign or value of the measure and the direction of inequality (if appropriate), and the extent of inequality represented by the minimum and maximum bounds of the measure. For results presented using a table or graph, this explanation could be included as a note (Panel 4).

**Table:** 

**Panel 4 Example explanations of the interpretation of units of measurement for difference and ratio**	
The following provide explanations of the interpretations of the pairwise summary measures difference, which is calculated as Subgroup A estimate – Subgroup B estimate, and ratio, which is calculated as Subgroup A estimate / Subgroup B estimate:	
The pairwise summary measure difference is expressed in the same unit of measurement as the health indicator. A value of 0 indicates no inequality and larger absolute values indicate higher levels of inequality. The direction of inequality is indicated by values above or below 0. The pairwise summary measure ratio is unitless and assumes only positive values. A value of 1 indicates no inequality, and the further the value is from 1, the higher the level of inequality. The direction of inequality is indicated by values above or below 1. Equivalent ratios can be expressed using their reciprocals (e.g. 2/1 = 2 the reciprocal of 1/2 = 0.5).	

##### B. Association and causation

Analyses and measures of health inequality describe associations between a health indicator and one or more dimensions of inequality [4, 7]. For example, an analysis may show that health outcomes are better among more socially advantaged population subgroups. Multiple regression can show whether a health indicator is associated with a dimension of inequality after adjustment for other dimensions of inequality. For example, multiple regression can indicate whether the prevalence of diabetes differs by educational attainment, adjusting for differences in age and sex. Compound vulnerability and advantage (assessments that quantify the extent to which certain demographic, socioeconomic and geographic conditions act together to confer vulnerability or advantage with regards to a particular health or health-related condition) take into account multiple dimensions of inequality at the same time. Such findings can link health and inequality dimension variables, establishing associations between them – but they do not support claims of causation (e.g. that change in education would raise or lower the risk of diabetes, holding correlates of both education and diabetes risk constant).

Decomposition analyses break down variation in a health indicator or its association with a dimension of inequality into the separate contributions of measurable characteristics (e.g. education, place of residence, socioeconomic status) to that variance or association [4]. Such analyses provide a statistical explanation of health inequality. They do not identify causes of differences in health without further assumptions.

Texts should avoid claiming that relationships between variables are causal, or interpreting them as such, unless the assumptions on which the causal inference rests are stated explicitly and they give credible and relevant contextual evidence [41]. Claims of causation require other conditions to be met, namely, temporality (i.e. inequality dimension characteristic precedes the health outcome and not vice versa) and non-confounding (the health outcome is not also caused by measured or unmeasured factors other than the inequality dimension). This is important to ensure that the findings (and their limitations) are communicated clearly and accurately.

When describing the relationship between health and dimensions of inequality, terms such as “linked with”, “associated with” or “correlated with” may be used to express correlation, while “determines” or “affects” convey causation. Even with careful word choices, however, readers may still infer causation from correlation statements [42]. An explicit statement is usually warranted to clarify that results do not support a causal interpretation.

In papers that report the results of descriptive health inequality measurements, the introduction section should clarify that the research question is descriptive (i.e. exploring associations) and not causal. Causal attribution may be appropriately addressed in the discussion section. Assertions of causal attribution generally draw from multiple forms of evidence and should be accompanied by discussion of the credibility of the cause-and-effect claims, and evaluation of the strength of the evidence. Guidance for reporting on association and causation is summarized in Panel 5.

**Table:** 

**Panel 5 Guidance for reporting on association and causation**	
• Clarify in the introduction whether the research question concerns a correlational or causal relationship and ensure that the study design is capable of delivering evidence consistent with the research question. • Ensure that reporting of associations does not suggest causation; consider including a statement to explicitly state this. • Avoid drawing policy implications in the discussion section that rest on a causal interpretation of the descriptive evidence.	

### Macro strategies

Building on the micro aspects of writing, macro perspectives address larger issues regarding how reports are structured and the type of information they contain.

#### Strategy 3. Adhere to reporting guidelines and best practices

Adhering to established reporting guidelines, where applicable, can improve the accuracy, clarity and methodologic transparency of journal articles [43]. The use of reporting guidelines promotes completeness of reporting and confers credibility within the scientific community. It can also facilitate the subsequent inclusion of study results in larger reviews, and the uptake of evidence for decision-making.

There are hundreds of reporting guidelines available that have applications for reporting on public health and medical topics (as listed on the EQUATOR Network website [44]), and many academic journals endorse specific ones. Several guidelines have particular relevance to health inequality reporting in academic and scientific contexts. For example:

• The equity extension of the Strengthening Reporting of Observational Studies in Epidemiology (STROBE) reporting guidelines, STROBE-Equity, details practices to improve the consistency of reporting of health equity in observational studies [45].

• The Sex and Gender Equity in Research (SAGER) guidelines encourage the complete and routine reporting of disaggregated data about sex and gender as part of research design, data analysis, results and interpretation [30].

• The CONSIDER statement contains consolidated criteria for strengthening reporting of health research involving indigenous peoples [46].

• The equity extension of Preferred Reporting Items for Systematic reviews and Meta-Analyses (PRISMA), PRISMA-Equity, provides guidance for reporting equity-focused systematic reviews [47].

• The equity extension to the Consolidated Standards of Reporting Trials (CONSORT) statement, CONSORT-Equity provides guidance for transparent reporting of intervention effects in randomized trails where health equity is relevant [48].

WHO has developed a collection of best practices for reporting about health inequalities that have broad applicability across diverse target audiences and reporting outputs [49–51]. Some of the best practices that pertain to writing include: tailor the type of information and its presentation to the purpose of reporting and the needs, interests and abilities of the audience; provide background information to situate why inequality monitoring was done and what it aims to accomplish; include descriptions of the indicators, inequality dimensions, data sources, analysis methods and interpretation, highlighting any pertinent limitations; report findings using the most straightforward and simple measures of inequality possible, while ensuring the conclusions are accurate and supported by the underlying data; and include comprehensive information about the results (see Panel 6). The scope of reporting for manuscripts about inequality analyses should include information about the latest situation of inequality (based on the most recent data included in the analysis) and, if feasible, change over time and benchmarking [4]. In considering the impact of the findings, the strength of the evidence on health inequalities (including issues related to data availability and health inequality monitoring capacity) can be linked to the strengths and limitations of the underlying health information system. Acknowledging this can help to promote a consistent focus on the information system strengthening that is necessary for improved health inequality monitoring over time.

**Table:** 

**Panel 6 WHO best practices for reporting on health inequalities: include key information about the results**	
The following best practices are recommended when reporting the results of health inequality analyses.	
• Report disaggregated health data in a straightforward and intuitive manner. In some cases, it may be sufficient to report only disaggregated data (and not summary measures of inequality) to present inequality analyses, especially if the scope of reporting is confined to the latest status of inequality in a limited number of indicators. • If reporting summary measures of inequality, include both absolute and relative measures (absolute measures of inequality express the magnitude of difference in health between subgroups, and relative measures express the proportional difference in health between population subgroups [4]). • Indicate the overall value of the health indicator in the whole population (e.g. the national average). • Indicate the population share of subgroups. • Flag results based on small sample sizes, if applicable. • Make code for data preSimpleParation and analysis available in a public repository. • Make data publicly available, if possible.	

### Meta strategies

Adopting a meta perspective, we considered the process of preparing written reports and how it can be enhanced through inclusive, equity-upholding practices. This perspective is focused on a larger scope than the micro and macro strategies discussed above, aiming to ensure that the act of reporting is in line with the broader goal of advancing health equity. Although the below strategy includes discussions of language choices and messaging (which might be considered as part of micro and macro perspectives, respectively), these issues are raised here to emphasize the collaborative process of arriving at language and messaging that is affirmative and strength based.

#### Strategy 4. Respect and uphold the interests of affected communities through inclusive writing practices

Throughout the writing process (as well as the preceding steps in the research process), there are opportunities – and obligations – to respect and uphold the interests of affected populations that are the focus of the inequality monitoring analyses and for whom equity-oriented actions seek to benefit. Public health and medical organizations have issued guidance for engaging with communities and partners to promote an inclusive approach to public health communications [36, 52]. Here, in line with the scope of this paper, we address specific considerations for inclusive practices that arise when preparing and writing academic manuscripts on health inequality measurements.

##### A. Affirming language

Language choices can both reflect and reinforce social norms, which in turn influence attitudes, actions and priorities within societies [53, 54]. Writing should aim to use affirming language that conveys the value and dignity of people and communities. The selection of appropriate and affirming language requires contextual knowledge and continual learning, as language preferences may differ between circumstances and evolve over time.

The use of person-first language – e.g. “person experiencing homelessness” rather than “homeless person” – is a widely-adopted language style that intends to acknowledge the humanity of individuals by “putting the person first” [55]. In some cases, however, identity-first language may be more acceptable by some or all members of the affected group. For example, in some contexts, “disabled person” is preferred to “person with a disability”, acknowledging that language preferences vary from person to person, and may change over time [55]. The onus is on writers and researchers to remain up to date with current language use and, when feasible, consult directly with the individuals and groups about their preferences^7^.

Likewise, careful and ongoing consideration should be given to the use of terms to describe population subgroups. Where possible, descriptive terms should be used to label subgroups based on their defining characteristic (e.g. rural or urban residents, richer or poorer subgroups). If subgroups reflect data that are based on self-determination, such as gender, ethnic and racial identities, this should be acknowledged. In some cases, it may be required to use general terminology to describe subgroups that experience disadvantage. Terms such as “disadvantaged”, “high-risk”, “left behind”, “marginalized”, “underprivileged”, “underserved”, “vulnerable” and “worse-off” are vague, and may imply that a condition is inherent to a group or unintentionally insinuate blame or inherent vulnerability [56]. Further, these terms may imply comparisons and require further qualification: e.g. worse off than whom and in what regard? At high risk for what and compared with whom? If such terms are used, the underlying assumptions and definitions should be made explicit (see examples in Panel 7).

**Table:** 

**Panel 7 Explanations about the use of terminology to describe situations of disadvantage**	
The WHO book *Health inequality monitoring: harnessing data to advance health equity* includes the following note about terminology [4]:	
Throughout the book, we have endeavoured to use language and terminology that reflect inclusivity and respect for all individuals and population subgroups. In our discussions of population subgroups, we acknowledge the inherent diversity within these groups, and the intersectional nature of the characteristics that influence and shape health experiences. When referring to subgroups that experience “disadvantage”, the intention is to recognize specific historic and systemic factors linked to relative social disadvantage, such as lower economic status or education level. This and other terminologies are not meant to convey negative stereotypes, stigmatization or blame.	
In their paper about reporting health equity considerations in cluster and individually randomized trials, Petkovic et al. include the following note about the use of the term “socially disadvantaged” [57]:	
Throughout this paper, we use the term “socially disadvantaged” to denote that people are disadvantaged by differences in distribution of power and resources which structure their living and working conditions and affect their opportunities for health. We recognize that this terminology may be seen as labeling or stigmatizing, which is not our intent. Some people and populations may prefer other terms to fit their context….	

Because the connotations associated with terminology vary across populations and change over time, its use bears continuous discussion and consideration. For example, the term “stakeholder” carries colonial connotations in some communities as its historical roots refer to the person who drove a stake into the ground to demarcate land they were claiming from Indigenous Peoples. An alternative term “interest-holders” has been proposed to describe “groups with legitimate interests in the health issue under consideration” [58]. As another example, the term “minoritized” is preferred in some contexts over terms such as “disadvantaged”. According to the report *Structural Racism*,* Ethnicity and Health Inequalities in London*, “minoritized” reflects social processes of power and domination, unlike “minority” which references numerical presence in a population [59]. In the context of that report, it is acknowledged that “minoritized” allows for the reality that some ethnic groups who are minorities in London are majorities in the global population.

##### B. Strength-based messaging

Reports about health inequality require sensitivity when discussing the poorer health outcomes or indicators of population subgroups in situations of social disadvantage. Often, academic reports emphasize the link between social disadvantage and poorer health, focusing on the size of the gap between subgroups. This may inadvertently stigmatize groups in vulnerable situations, reinforce power imbalances, erode trust and credibility with communities and shift the focus away from the structural factors that underlie inequalities [4]. Messaging should avoid discourse that represents people in terms of deficiencies, instead aiming to present an asset-based analysis, contrasting the disadvantageous situation of the individuals or communities with their strengths.

Strengths-based approaches to reporting on health inequalities convey language and solutions aligned with overcoming an issue, shifting away from a focus on “absence, lack or failure” to a focus on resilience and strengths [60]. Thurber et al. have demonstrated approaches to support strengths-based quantitative analysis in a study involving Indigenous children in Australia. Strategies such as identifying factors that are protective against negative outcomes and identifying factors associated with positive health outcomes enable a more positive story to be told, while maintaining statistical rigour [61] (see example in Panel 8).

**Table:** 

**Panel 8 Examples of deficit and strength-based messaging approaches**	
In a case study of Aboriginal and Torres Strait Islander child wellbeing, Thurber et al. (2020) contrasted three approaches to describing the relationship between child mental health and caregiver employment status.	
• A deficit approach described: “poor-moderate mental health is significantly more common among children whose caregivers are not employed versus employed.” • A protective factors approach described: “poor-moderate mental health is significantly less common among children whose caregivers are employed versus not employed.” • A positive outcome approach described: “good mental health is significantly more common among children whose caregivers are employed versus not employed.”	
The authors emphasized the merits of the strengths-based approaches, which more closely reflected the community’s values and principles and were more likely to support positive changes [61].	

##### C. Engaged writing processes

As part of the writing process, engagement with the views of local experts, communities and those with lived experiences can result in a richer understanding and appreciation of a diversity of perspectives and range of experiences. This may require an openness to question established beliefs and notions. Just as written outputs may be piloted with their intended audience, they may also be piloted with affected populations to promote their acceptability, transparency and impact. This can help to ensure that writing avoids potential sensitivities and pejoratives, and that it does not perpetuate stigma, discrimination, or recrimination against disadvantaged groups. Further, engagement with practitioners (including health and social service providers, health system programmers and planners and policymakers) can enrich the understanding and interpretation of the data. Co-authoring written outputs with members of the communities represented in the research is a good practice that is increasingly encouraged (and sometimes required) by academic journals.

## Emerging issues and reflections

Well-written reports can be instrumental in highlighting health inequalities, bringing attention to key issues, and prompting important changes in policy, programmes and practices. While existing literature has provided guidance for scientific writing in general, this paper fills a gap by focusing on micro, macro and meta issues that arise in reporting the results of health inequality analyses.

The impact of health inequality research is shaped by the multidisciplinary nature of the field. On the one hand, this underscores the broad relevance of health inequality research across diverse audiences and disciplines. On the other hand, the impact of health inequality research may be diminished by the fragmented, siloed and multi-disciplinary nature of the field, and stands to be enhanced through efforts to further promote connectivity [1, 18]. We believe that establishing common guidance for writing about the results of health inequality analyses may be part of this larger effort to augment the impact of this research. Given the growing output of research involving health inequality measurements, the inclusion of a greater number of articles in systematic reviews and meta-analyses is one concrete benefit of more standardized approaches to reporting.

The use of “micro-macro-meta” framing facilitates consideration of a wide range of issues. The strategies offered across these three levels encourage a dynamic and thorough approach to preparing impactful written reports. Of particular importance in writing about health inequality is the inclusion of the “meta” component. Traditional writing guidance, peer review and editorial feedback often tend to focus on the micro and macro aspects of writing. The addition of the meta – that is, the processes of planning and carrying out writing projects – prompts more reflexive consideration of issues, including those related to engagement and collaboration. This component holds the potential to promote impact through the written output itself by ensuring that the language and messaging are grounded in the realities of the wider audiences and populations. The writing process itself can also be an opportunity to recognize and uphold values that are often aligned with advancing health equity, such as inclusivity, empowerment and respect.

The preparation of this paper was an opportunity for the author group to reflect upon and strengthen guidance for writing about health inequality evidence, and to consider other aspects of health inequality monitoring that require further attention. Writing is part of the larger, interlinked cycle of health inequality monitoring, which encompasses: (a) determining the scope of monitoring; (b) obtaining disaggregated data; (c) analyzing data; (d) reporting results; and (e) translating knowledge into action [49]. Within this framing, writing is one aspect of (d) reporting results, though is linked to the other parts of the cycle. For instance, the ability to write clearly about data analysis techniques requires familiarity with the underlying data sources and approaches. Acknowledging that ambiguity in writing may stem from ambiguity in the underlying concepts, there is an ongoing need to strengthen capacity across all parts of the health inequality monitoring cycle [4].

Written reports and outputs, which themselves may take diverse forms, are often accompanied by other communication products, such as interactive data visualizations, videos and podcasts. We acknowledge the need for guidance to promote the accuracy and accessibility of other forms of communication about health inequalities – and to address issues that were outside the scope of this paper, including the preparation of data tables and visuals [62].

The expanding use of artificial intelligence (AI)-assisted tools as part of writing processes presents unique considerations, opportunities and uncertainties for those writing about health inequality measurements. Guidance from journals and professional bodies about the use of these tools, and disclosure requirements, is evolving as the tools become more advanced and entrenched in writing and publishing processes [63]. We highlight the positive possibilities of using such tools for editorial purposes to promote equity in the writing process in the field of health inequality research (i.e. making supports available to writers who may not otherwise have access, and/or easing linguistic disparities) [64]. Yet, the full picture of how AI-tools are being utilized and their limitations (e.g. perpetuating biases, plagiarism, data ownership issues and inaccuracy [65, 66]) remains an area for further study.

## Conclusions

In conclusion, this paper has offered four strategies for strengthening the accuracy and impact of writing about the results of health inequality analyses, addressing the deliberate and consistent use of terminology, the presentation of statistical content, adherence to reporting guidelines and best practices and respecting and upholding the interests of affected communities. Under these strategies, we highlighted key issues and common sources of confusion in health inequality reports, as well as practical guidance and examples. The paper underscores the importance of considering multiple aspects of writing and the writing process as part of larger aims related to advancing health equity through evidence on health inequalities.

## Data Availability

No datasets were generated or analysed during the current study.

## Footnotes

1 From a grammatical standpoint, it may be more accurate to indicate “economic status-related inequality” when referencing wealth, income, asset or other dimensions of inequality related to the economic situation of an individual, household, or other unit; however, this amount of text is cumbersome to report. We note that this framing is not substantively different from saying “economic-related inequality,” and recommend that the latter, shorter formulation be used.

2 Given that these different characteristics may lead to distinct health needs, identification of sex-related inequalities in health do not necessarily indicate health inequities. However, where such differences cannot be explained by biological determinants, they are usually understood to be driven by gender inequality. Gender inequality refers to unequal capacities and opportunities to access and control all social, economic and political resources (e.g. health services, education, voting rights) and is necessarily unjustifiable [4].

3 There are some scholars who explicitly propose this convention to acknowledge that sex and gender interact. The Stanford resource, Gendered Innovations in Science, Health & Medicine, Engineering, and Environment, contains further definitions, examples and discussion regarding “sex” and “gender” [32].

4 Article 1 of the United Nations Educational, Scientific and Cultural Organization (UNESCO) 1978 Declaration on Race and Racial Prejudice states that “all human beings belong to a single species and are descended from a common stock. They are born equal in dignity and rights and all form an integral part of humanity” [33].

5 This is because viewing race as biological tends to reinforce prejudicial notions of a group as inferior and it directs attention away from the need to improve the conditions in which people live in order to improve their health and narrow inequalities.

7 Such consultations may not always be feasible, for example, if reporting the results from a large multi-country analysis. Researchers are encouraged to consult with literature, recommendations and guidance issued by relevant professional organizations and citizen groups.

## Declarations

### Authors Information

NB, KK, DN, AS, ARH are part of the Health Inequality Monitoring team at WHO Headquarters. The team has authored and contributed to books on health inequality monitoring [4, 67, 68], and published global and national health inequality reports, and journal articles, including systematic reviews [69]. It conducts health inequality monitoring courses and workshops with diverse audiences and leads the development of journal special issues devoted to reporting on health inequalities [70–73]. Also working at WHO Headquarters, TSK leads work on assessing barriers to effective coverage with health services and mainstreaming equity approaches into health programming. OJM has over 25 years of experience as the Senior Regional Advisor in Social Epidemiology and Health Equity at the Pan American Health Organization. AJDB, director of the International Center for Equity in Health, is a leading researcher and author in the field of global health equity and has contributed to making equity analyses more accessible to a wider audience. PB has been a leader globally on the concepts of health inequalities and health equity and the implications for measurement. PG has contributed to national and international projects that review and monitor the social determinants of health inequities. OOD has coauthored a handbook on health equity analysis with survey data [25]. VW has led reporting guidelines on assessing health equity in systematic reviews, randomized trials and guidelines [47, 48, 74].

## Abbreviations

CONSIDER: CONSolIDated critERtia for strengthening the reporting of health research involving Indigenous Peoples
CONSORT: Consolidated Standards of Reporting Trials
EQUATOR: Enhancing the QUAlity and Transparency Of health Research
HIV/AIDS: Human immunodeficiency virus / Acquired immunodeficiency syndrome
PRISMA: Preferred Reporting Items for Systematic reviews and Meta-Analyses
SAGER: Sex and Gender Equity in Research
STROBE: Strengthening Reporting of Observational Studies in Epidemiology
UNESCO: United Nations Educational, Scientific and Cultural Organization
WHO: World Health Organization
